# Effect of Iron Concentration on the Co-Production of Fucoxanthin and Fatty Acids in *Conticribra weissflogii*

**DOI:** 10.3390/md22030106

**Published:** 2024-02-24

**Authors:** Ke Peng, David Kwame Amenorfenyo, Xiangyu Rui, Xianghu Huang, Changling Li, Feng Li

**Affiliations:** College of Fisheries, Guangdong Ocean University, Zhanjiang 524088, China; 2112101138@stu.gdou.edu.cn (K.P.); davidamenorfenyo@yahoo.com (D.K.A.); 2112001098@stu.gdou.edu.cn (X.R.); huangxh@gdou.edu.cn (X.H.); licl@gdou.edu.cn (C.L.)

**Keywords:** *Conticribra weissflogii*, iron, fucoxanthin, fatty acids

## Abstract

The production of fucoxanthin and fatty acids in *Conticribra weissflogii* has been examined, but there is still a lack of understanding regarding the impact of trace elements, including iron, on their co-production. To address this knowledge gap, this study investigated the effects of FeCl_3_·6H_2_O on the growth, fucoxanthin, and fatty acids of *C. weissflogii*. The findings revealed that the highest cell density (1.9 × 10^6^ cells mL^−1^), cell dry weight (0.89 ± 0.15 g L^−1^), and total fatty acid concentration (83,318.13 µg g^−1^) were achieved at an iron concentration of 15.75 mg L^−1^, while the maximum carotenoid and fucoxanthin contents were obtained at an iron concentration of 3.15 mg L^−1^. The study demonstrated that the content of the active substance in *C. weissflogii* could be increased by adjusting the iron concentration, providing new information as to the more efficient co-production of fucoxanthin and fatty acids and offering experimental support for large-scale production.

## 1. Introduction

Microalgae, as the main primary producers in marine ecosystems [1], are diverse, widely distributed, and rich in a variety of bioactive substances [2]. These microorganisms play a vital role in the material cycle and energy flow, and have become a valuable resource for commercialization in recent years [3,4]. Their abundant unsaturated fatty acids, proteins, lipids, and carotenoids (including docosahexaenoic acid, eicosapentaenoic acid, β-carotene, astaxanthin, lutein, and fucoxanthin) [2] make them a promising source of biological resources for the food, bait, cosmetics, health care products, and pharmaceutical industries [5,6,7].

Fucoxanthin is a type of carotenoid, found in brown algae, diatoms, and golden algae, and its unique molecular structure makes it effective in terms of anti-obesity, tumor inhibition, anti-inflammatory, anti-diabetic, cancer prevention, and especially antioxidant effects [8,9,10,11]. It has been found that the content of fucoxanthin in microalgae is more than four times that in macroalgae [12]. Although the production of fucoxanthin and fatty acids in *C. weissflogii* has been studied, there is still a gap in our understanding of the influence of trace elements, such as iron, on their co-production.

Some diatoms are rich in eicosapentaenoic acid (EPA), docosahexaenoic acid (DHA), and other highly unsaturated fatty acids in addition to fucoxanthin. Studies have shown that EPA and DHA can improve the survival rate and growth performance of aquatic animals and provide a broad market for human biomedicine [13]. Currently, marine fish are the main source of the essential fatty acids, EPA, and DHA. However, due to rising fish prices and overfishing, diatoms are being considered a promising alternative for the large-scale production of EPA and DHA products, as they can be easily controlled in their growth environment [14].

*Conticribra weissflogii* is a typical diatom that is easy to culture, has a fast growth rate, and is considered a potential cell factory for the production of fucoxanthin and unsaturated fatty acids [15,16]. Iron is an essential trace element for microalga growth, enzymatic reactions, nitrogen metabolism, and chlorophyll synthesis [17], and iron deficiency or excess inhibits the productivity of microalga cells [18,19]. In addition, the concentration of iron can fluctuate significantly [20], potentially affecting the growth rate of microalgae and the synthesis of secondary metabolites, including fucoxanthin and fatty acids. In controlled cultivation, the optimization of iron levels can be a crucial factor for enhancing productivity and cost-efficiency. However, the specific effects of varying iron concentrations on fucoxanthin and fatty acid co-production in *C. weissflogii* remain poorly understood. This lack of knowledge constrains the ability to effectively harness the full potential of this microalga for the commercial production of these valuable compounds.

The present study aimed to address this gap by systematically investigating the effect of different iron concentrations on the growth dynamics of *C. weissflogii* and the concurrent production of fucoxanthin and fatty acids. Understanding this relationship can enhance our understanding of the metabolic flexibility of this microalga and inform optimization strategies for industrial cultivation processes, ultimately contributing to the sustainable production of high-demand bioactive compounds.

## 2. Results

### 2.1. Growth of C. weissflogii under Different Iron Concentrations

**Figure 1:**
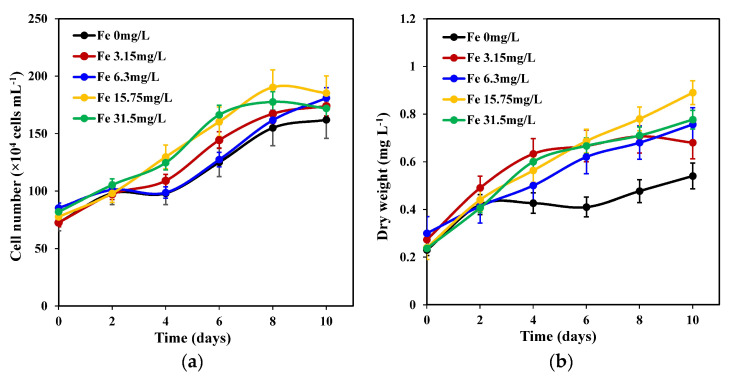
Changes in cell number (**a**) and dry weight (**b**) of *C. weissflogii* cultures (mean ± SD, *n* = 3).

As shown in Figure 1a, varied iron concentrations demonstrated different effects on *C. weissflogii*. Although each group exhibited a similar growth pattern between day 2 and day 10, those with 15.75 mg L^−1^ and 31.5 mg L^−1^ iron concentrations showed remarkable growth on day 8 compared with the other three groups, which continued to grow until they reached their maximum growth on day 10. A 15.75 mg L^−1^ iron concentration showed a maximum growth of 1.9 × 10^6^ cells mL^−1^ on day 10 compared with other treatments. However, there was no significant difference (*p* < 0.05) between the other iron treatment groups, except for that with a 0 mg L^−1^ iron concentration treatment.

The maximum biomass concentrations (dry weight) of the five iron concentration groups were obtained on the 10th day of continuous growth (Figure 1b). Specifically, treatment groups with a 15.75 mg L^−1^ iron concentration obtained the highest biomass concentration compared with other iron concentration treatment groups, and this was significantly higher than that of the 0 mg L^−1^ iron concentration group. No significant differences were observed between biomass concentrations of 15.75 mg L^−1^ iron, 3.15 mg L^−1^, 6.3 mg L^−1^, and 31.5 mg L^−1^ iron concentrations.

### 2.2. Changes in the Pigment Content of C. weissflogii under Different Iron Concentrations

**Figure 2:**
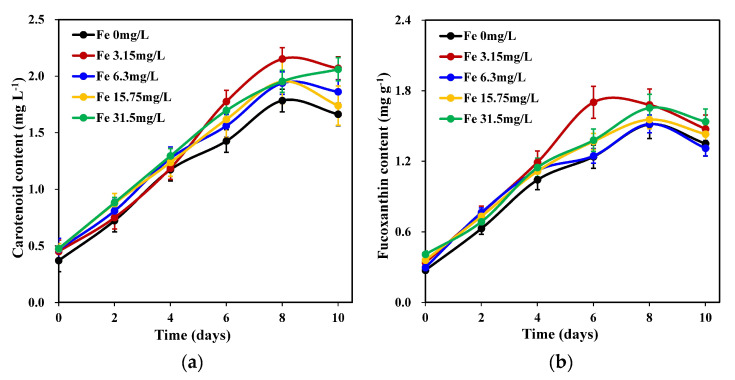
Changes in carotenoid content (**a**) and fucoxanthin content (**b**) of *C. weissflogii* cultures (mean ± SD, *n* = 3).

Iron concentration had a significant effect on carotenoid accumulation in *C. weissflosii* (*p* < 0.05). The results (Figure 2a) showed that of the carotenoid accumulation of the 0.00 mg L^−1^ treatment group was significantly lower than that of the other treatment groups. The 3.15 mg L^−1^ iron concentration groups obtained the maximum carotenoid content on day 8, which was significantly higher than that of the other groups (*p* < 0.05).

As shown in Figure 2b, the fucoxanthin concentration of each iron concentration group first increased (from day 2 to day 8) and then decreased (after day 8). The 3.15 mg L^−1^ iron concentration resulted in the maximum fucoxanthin concentration on the 6th day, which was the highest among all treatment groups. However, no significant differences were observed between the experimental groups (*p* > 0.05).

### 2.3. Effects of Different Iron Concentrations on C. weissflosi Biomass and Fucoxanthin Productivity

**Figure 3:**
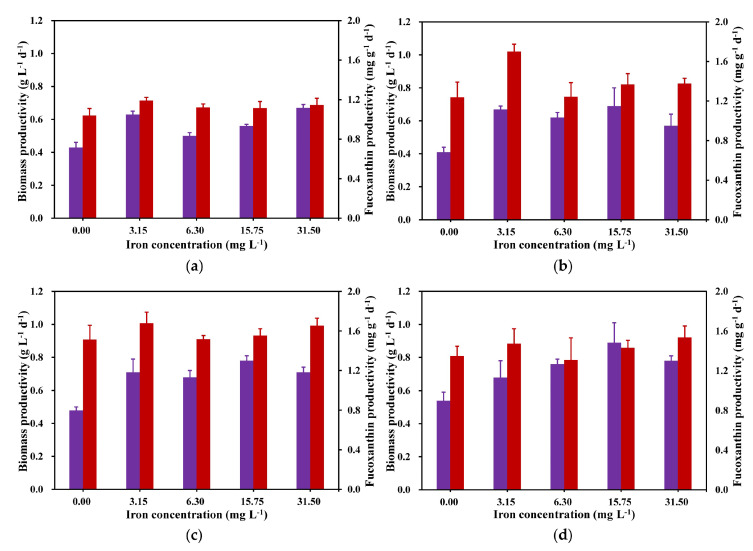
Biomass (purple) and fucoxanthin (red) productivity on day 4 (**a**), day 6 (**b**), day 8 (**c**), and day 10 (**d**) (means ± SD, *n* = 3).

Biomass and fucoxanthin productivity on the 4th, 6th, 8th, and 10th days of cultivation were selected for comparative analysis. As depicted in Figure 3, there were significant differences in biomass and fucoxanthin productivity on other days, except for fucoxanthin productivity on day 4. The maximum biomass productivity was observed in the later part of the cultivation period (day 10), while the maximum fucoxanthin productivity was attained on day 6 of the cultivation period. From Figure 3, except for that on day 4, the 15.75 mg L^−1^ iron concentration showed the highest biomass productivity (Figure 3b) compared with that of all other iron concentration treatment groups, and except for the 10th day, the highest fucoxanthin yield in each experimental group was obtained by the group with an iron concentration of 3.15 mg L^−1^, so it can be determined that the biomass accumulation of *C. weissflosi* is more suitable for an iron concentration of 15.75 mg L^−1^, and the accumulation of fucoxanthin is more suitable at a 3.15 mg L^−1^ iron concentration.

### 2.4. Fatty Acid Composition and Concentration of C. weissflosi at Different Iron Concentrations

As shown in Table 1, 30 fatty acids were measured under different iron concentrations, including 12 unsaturated fatty acids (UFAs) and 18 saturated fatty acids (SFAs). The main fatty acids were C16:1n7, C20:5n3, C22:6n3, C14:0, and C16:0. Except for that of the group with an iron concentration of 0 mg L^−1^, the UFA content of each group was higher than that of SFAs, and the UFA content of the group with an iron concentration of 15.75 mg L^−1^ was higher than that of the other groups. The order of fatty acid content from highest to lowest was SFA (40.9–60.4%) > MUFA (monounsaturated fatty acid) (36.9–39.1%) > PUFA (polyunsaturated fatty acid) (18.0–20.2%).

**Figure 4:**
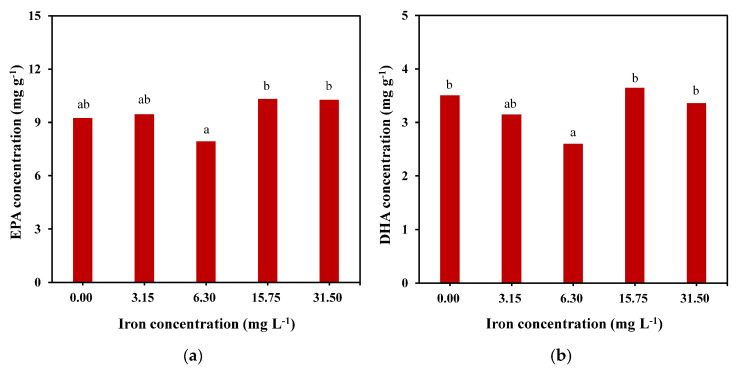
EPA (**a**) and DHA (**b**) concentrations of *C. weissflogii* under different iron concentration culture conditions. Means within the same column of different letters are significantly different at (*p* < 0.05).

Under the condition of 15.75 mg L-1 of iron, the concentrations of C14:0, C16:1n7, C16:0, EPA, and DHA were higher than those in the other groups, and the total lipid content reached the cumulative maximum. As shown in Figure 4, the EPA concentration of the 15.75 mg L^−1^ group reached 10.3 mg g^−1^ (approximately 12.4% of the total lipids), which was slightly higher than that of the 31.5 mg L^−1^ group and significantly higher than that of the other groups. It was also found that under the same iron concentration of 15.75 mg L^−1^, the DHA concentration reached the maximum value of approximately 3.6 mg g^−1^, accounting for 4.4% of the total lipid content.

## 3. Discussion

Iron is one of the important trace elements necessary for cell growth metabolism and lipid synthesis in microalgae and is also a limiting factor involved in a variety of enzymatic reactions and transport systems of microalgae, such as redox reactions, oxygen carrier proteins, photosystem II, nitrogen depletion, and chlorophyll synthesis [17,21,22,23]. Different concentrations of iron ions have different effects on the growth of microalgae, and iron concentrations that are too high or too low will limit the growth and lipid synthesis of microalgae because iron is the carrier of certain oxidoreductase enzymes, while the components of coenzymes in algal cells and iron deficiency can affect a variety of metabolic processes to inhibit cell growth and lipid synthesis [18,19,24]. Liang et al. [25] pointed out that the fastest growth rate and the highest biomass were observed in *Phaeodactylum tricornutum* at a concentration of 0.5 mg L^−1^, and the highest content of total lipids was observed at a concentration of 0.25 mg L^−1^. The total lipid content of this alga increased with an increase in the concentration of iron before reaching the optimal concentration of iron, and the synthesis of the total lipids of the microalgae was inhibited when the concentration of iron exceeded the optimal concentration. It has also been noted that iron concentrations as high as 2 mg L^−1^ have positive effects on most microalgal species, but negative effects are usually observed when iron concentrations exceed 40 mg L^−1^ [26]. Zhou et al. [27] reported that the optimal iron concentration for the growth of *Microcystis aeruginosa* ranged from 3 to 12 mg L^−1^. When the Fe concentration exceeded 12 mg L^−1^, the concentration of Fe ions in the algal cells increased significantly, resulting in the inhibition of algal growth. Zhou et al. reported that the utilization of iron by *M. aeruginosa* was significantly correlated with its growth status in the optimal concentration range. In this experiment, the cell biomass and fatty acid content of *C. weissflogii* reached the maximum value at an iron concentration of 15.75 mg L^−1^, indicating that the range of iron concentrations required for growth and lipid synthesis varied among different algal species.

Iron ions can enhance the pigment synthesis and cell membrane stability of microalgae at appropriate concentrations to improve the accumulation ability of active substances [24,28,29,30]. Wu et al. [31] found that adding an appropriate concentration of ferric chloride during the culture of *Navicula tenera* can significantly promote the accumulation of pigments in *N. tenera*. In another study [32], it was shown that iron-enriched medium stimulated fucoxanthin accumulation in *Nitzschia* sp. and *Nanofrustulum shiloi*. Zhu et al. [33] observed that at an iron concentration of 0.135 mg L^−1^, the carotenoid content of *P. tricornutum* showed an increase of 5% to 30% compared to that under iron deficiency. In the results of a study [34] on the qualitative and quantitative effects of Fe concentration on the pigment composition of *P. tricornutum*, it was found that both the β-carotenoid and fucoxanthin contents of *P. tricornutum* reached a maximum und a 10 μM Fe concentration; however, a strong decrease in fucoxanthin content was observed in *P. tricornutum* grown under very low Fe concentrations (0.001 and 0.01 μM). Kosakowska et al. [34] suggested that *P. tricornutum* prioritizes the accumulation of diadinoxanthin at the expense of fucoxanthin synthesis to compensate for the sharp decrease in the content of β-carotenoids (another photoprotective pigment) under low-Fe conditions. In the present study, the contents of carotenoids and fucoxanthin showed a trend of increasing and then decreasing in all groups, which may be attributed to the increase in cell density in the later stages of cultivation and the inter-cellular shading effect that reduces the uptake of light energy, which in turn reduces the rate of synthesis of carotenoids and fucoxanthin. In addition, the maximum values of the carotenoid and fucoxanthin contents of *C. weissflogii* were found in the 3.15 mg L^−1^ treatment group, suggesting that this iron concentration may be the optimal concentration for the accumulation of carotenoids and fucoxanthin in *C. weissflogii*. In addition, we observed that the carotenoid content of *C. weissflogii* was the lowest when iron was deficient, indicating that iron deficiency could inhibit the accumulation of carotenoids in *C. weissflogii* to some extent.

Fatty acid double bonds are formed by desaturase, which presents three conserved histidine clusters that are bound to Fe^2+^ to form the active center of the enzyme [35]. Therefore, the concentration of iron may affect the fatty acid composition of microalgae by influencing desaturase activity. Liang et al. [25] found that a high concentration of FeSO_4_ (1 mg L^−1^) was conducive to the synthesis of EPA, DHA, and PUFA in *P. tricornutum*. However, another study on the effect of iron on the fatty acid composition of *Tropidoneis maxima* [36] showed a decreasing trend for EPA, DHA, and PUFA in *T. maxima* and the influence of high concentrations of FeSO_4_ (1 mg L^−1^). Jiang observed [37] that the PUFA content of *Isochrysis galbana* decreased under Fe^3+^ concentrations above and below 24.5 μM, while the DHA content of *I. galbana* decreased under Fe^3+^ concentrations above and below 60.5 μM. These studies indicate that the effect of iron on the fatty acid composition of microalgae is species-specific. In this study, EPA, DHA, and PUFA contents were highest at an iron concentration of 15.75 mg L^−1^; an iron concentration above or below this could inhibit polyunsaturated fatty acid synthesis in *C. weissflogii*. In addition, the SFA content was significantly higher than the PUFA content under an iron concentration of 0 mg L^−1^, which may be due to the lack of Fe^3+^ in the medium, resulting in the inability of the conserved histidine clusters of the fatty acid desaturase to bind with sufficient Fe^3+^ to form the active center of the enzyme, thus affecting the process of saturated fatty acid desaturation.

## 4. Materials and Methods

### 4.1. C. weissflogii Strain and Culture Conditions

The *C. weissflogii* strain [15] used in this study was isolated from a shrimp pond in Southern China and preserved at the Laboratory of Algae Resource Development and Aquaculture Environmental Ecological Restoration of Guangdong Ocean University. This strain was grown autotrophically at a temperature of 25 °C in a modified F/2 medium, which contained NaNO_3_ (75 mg), KH_2_PO_4_ (5 mg), Na_2_SiO_3_-9H_2_O (20 mg), F/2 trace element solution (1 mL), and F/2 vitamin solution (1 mL) per liter of double-distilled water. The F/2 trace element solution comprised C_10_H_14_N_2_Na_2_O_8_ (4160 mg), FeCl_3_·6H_2_O (3150 mg), MnCl_2_·4H_2_O (180 mg), ZnSO_4_·4H_2_O (22 mg), CuSO_4_·5H_2_O (10 mg), H_4_MoNa_2_O_6_ (6 mg), and CoCl_2_·6H_2_O (4160 mg) per liter of double-distilled water. The F/2 vitamin solution was formulated with biotin (0.5 mg), vitamin B_12_ (0.5 mg), and vitamin B_1_ (100 mg) per liter of double-distilled water. The *C. weissflogii* culture was maintained under a continuous light intensity of 30 μmol m^−2^ s^−1^ and mixed with continuous aeration in 5 L flasks with filtered seawater added to the F/2 culture medium.

### 4.2. Experimental Setup

In this experiment, the effect of Fe (FeCl_3_·6H_2_O) was evaluated using varying concentrations from 0 mg L^−1^ to 31.5 mg L^−1^. FeCl_3_·6H_2_O was firstly configured into a mother liquor with a concentration of 3.15 g L^−1^. For the experiment, 0 mL, 1 mL, 2 mL, 5 mL, and 10 mL of FeCl_3_·6H_2_O mother liquor were added to the iron-free f/2 medium to represent the 0-fold iron, 1-fold iron, 2-fold iron, 5-fold iron, and 10-fold iron treatment groups, respectively. The volume of the mother cultures was 700 mL with the initial inoculation density of approximately 6 × 10^5^ cells mL^−1^. The experiment was performed under LED light (30 ± 2 μmol m^−2^ s^−1^), aeration (0.4 L min^−1^), temperature (25 ± 2 °C), pH (8.0 ± 0.2), and salinity (25), and cultured in a 1 L glass cylindrical photobioreactor (inner diameter, 5 cm) for 10 days. All cultures were mixed via continuous bubbling with 100% filtered air. Illumination was provided form the side by a T8 LED Tube light (white) with a light–dark cycle of 24 h: 0 h for 10 days. All treatments were carried out in triplicates.

### 4.3. Analytical Methods

After each sampling, the total cells of *C. weissflogii* were counted using a Neubauer improved cell counting chamber (25 mm × 16 mm) under an Olympus BX53 light microscope.

Dry weight (DW) was determined by filtering a 10 mL of the algal suspension through a pre-weighted (M_1_) acetate membrane (47 mm, nominal pore size 1 um). The algal biomass was rinsed twice with 0.5 M ammonium bicarbonate. The filter membrane was placed in an oven and dried at 80 °C to a constant mass, and the total mass M_2_ was measured and recorded. DW is calculated using Equation (1):

(1) $$DW=\left(M_{2}-M_{1}\right)/10$$

The carotenoid content was determined via the ethanol extraction method [38,39]. Algal cells were collected via centrifugation (5000 rpm for 10 min). Then, after addition of a 95% volume fraction of ethanol to the algal cells (10 mL), the mixture was treated under dark conditions for 24 h. The supernatant was collected via centrifugation (5000 rpm, 10 min), and the optical density values (D) of the supernatant at 480 nm, 510 nm, and 750 nm were measured using a spectrophotometer. The carotenoid content was calculated using Equation (2):

(2) $$\rho \left(Carotenoids\right)=7.6\times \left[\left(D_{480}-D_{750}\right)-1.49\times \left(D_{510}-D_{750}\right)\right]$$

Fucoxanthin content was measured using an organic solvent extraction method [40]. *C. weissflogii* cells (80 mL) were collected via centrifugation (5000 r min^−1^, 10 min) at 4 °C and placed in a freeze dryer for 2 days. The freeze-dried algal cells were ground into a powder, then anhydrous ethanol was added so that the material-to-liquid ratio was 1 g:40 mL and extracted twice, each time for 1 h, in the dark at 60 °C. The supernatant was collected via centrifugation at 5000 r min^−1^ for 10 min, and then the absorbance was measured at 445 nm using a UV spectrophotometer (D_445_). The fucoxanthin content was calculated using Equation (3):

(3) $$C\left(Fucoxanthin\right)=\left(1000\times D_{445}\times N\times V\right)/\left(A^{\prime }\times M\times 100\right)$$

N is the dilution ratio; V is the volume of the crude extract; A′ is the theoretical absorption value of a solute, which is 1600; M is the sample mass.

The fatty acid composition was determined via gas chromatography [41]. The appropriate amount of the sample was weighed in a glass tube, 0.5 mol L^−1^ of NaOH methanol solution, shocked uniformly, was added and placed in a 60 °C heated water bath for 20 min of saponification; after sufficient saponification, it was removed and cooled. Boron trifluoride methanol complexing solution, shocked uniformly, was added to a 60 °C water bath for 6 min for methylation; after cooling, isooctane was added for extraction and filtered with a 0.45 nm organic filtration membrane, and the supernatant was put into the injection vials for determination.

The prepared samples were analyzed with an Agilent 7890A gas chromatograph with the parameters and measurement procedures described in the authors’ previous studies [15]. A single fatty acid methyl ester standard solution and a fatty acid methyl ester mixed standard solution were injected into the gas chromatograph, and the peaks were characterized. The parameters were as follows: a capillary column (DB-23MS, column length 60 m; internal diameter 0.25 mm; film thickness 0.15 μm), the split injection mode, a split ratio of 35:1; nitrogen as the carrier gas, an inlet temperature of 270 °C, an initial temperature of 100 °C, a duration of 13 min, temperatures of 100 °C~180 °C at a heating rate of 10 °C min^−1^ for 6 min, temperatures of 180 °C~200 °C at a heating rate of 1 °C min^−1^ for 20 min, temperatures of 200 °C~230 °C at a heating rate of 4 °C min^−1^ for 10.5 min, and FID as the detector. Under the above chromatographic conditions, the fatty acid standard solution and the sample solution were injected into the gas chromatograph and quantified according to the peak area of the chromatogram. Sensitivity analysis was used to check the robustness of the fatty acid concentration.

Statistical analysis was performed using the SPSS for Windows statistical software package (IBM SPSS v26.0; Chicago, IL, USA). One-way analysis of variance (ANOVA) was used to test for significant differences (*p* < 0.05) between treatments, with the results presented as the mean ± SD (standard deviation).

## 5. Conclusions

This study demonstrated that the co-production of fucoxanthin and fatty acids in *C. weissflogii* could be considerably enhanced by adjusting the iron concentration in the growth media. These findings indicate that an optimal iron concentration of 15.75 mg L^−1^ is beneficial for achieving the highest cell density and total fatty acid content, while a different optimal iron concentration of 3.15 mg L^−1^ resulted in the highest carotenoid and fucoxanthin production. These results provide a foundation for the advancement of large-scale microalgae cultivation, particularly for the efficient and targeted production of valuable bioactive substances. This study adds to the broader understanding of microalgal biotechnology and highlights the potential of microalgae as a sustainable source of health-promoting compounds for aquatic organisms and humans. Furthermore, this study offers a valuable experimental framework and support for large-scale industrial production, which could have significant implications for nutrition, aquaculture, and the development of functional foods and nutraceuticals.

## Figures and Tables

**Table 1:** Fatty acid composition and concentrations (µg g^−1^) of *C. weissflogii* under different iron concentrations.

Test Items	0 mg/L	3.15 mg/L	6.3 mg/L	15.75 mg/L	31.5 mg/L
C4:0	18.13	19.57	45.97	15.83	43.11
C6:0	30.33	137.49	30.65	23.48	102.16
C8:0	263.9	112.91	119.47	130.3	269.79
C10:0	384.5	25.29	254.38	310.88	326.63
C11:0	Not detected	25.95	Not detected	35.37	53
C12:0	48.36	45.24	34.13	42.32	40.47
C13:0	Not detected	47	Not detected	Not detected	Not detected
C14:0	8321.98	8272.94	7789.73	8754.77	8622.26
C14:1n5	161.41	154.7	134.28	179.01	159.08
C15:0	950.01	1120.68	1055.68	1224.67	1180.82
C16:0	14,727.31	16,135.16	14,813.51	16,884.4	15,317.65
C16:1n7	22,365.49	25,421.25	24,049.79	26,828.85	24,082.01
C17:0	32.67	20.12	62.36	67.49	55.43
C18:0	501.14	425.01	338.4	470.36	367.42
C18:1n9c	999.17	1013.63	864.56	1102.62	988.37
C18:2n6c	281.33	260.08	198.92	252.58	264.04
C18:3n6	Not detected	170.72	60.06	20.83	27.57
C18:3n3	119.37	78.65	76.48	91.64	78.98
C20:0	57.75	69.59	92.43	126.45	85.36
C20:2	64.61	Not detected	101.77	Not detected	Not detected
C20:3n6	112.49	Not detected	66.83	Not detected	Not detected
C21:0	134.28	121.97	98.69	112.28	138.49
C20:3n3	126.25	Not detected	59.34	164.18	145.83
C20:4n6	123.17	86.67	78.95	97.39	114.95
C20:5n3	9254.11	9465.2	7936.26	10,328.37	10,280.99
C22:0	119.21	107.57	79.92	118.49	108.39
C22:6n3	3511.2	3146.65	2600.33	3647.48	3364.62
C23:0	318.16	Not detected	447.67	Not detected	Not detected
C24:0	1035.7	888.02	634.48	1025.92	967.25
C24:1n9	167.1	37.87	93.67	146.45	28.88
SFA	38,745.22	27,574.51	25,897.47	40,458.73	27,678.23
MUFA	23,693.17	26,683.25	25,142.3	28,256.93	25,258.34
PUFA	13,592.53	13,152.17	11,178.94	14,602.47	14,276.98
UFA	37,285.7	39,835.42	36,321.24	42,859.4	39,535.32
FA	76,030.92	67,409.93	62,218.71	83,318.13	67,213.55

## Data Availability

Data are contained within the article.
